# Optimisation of low and ultra-low dose scanning protocols for ultra-extended field of view PET in a real-world clinical setting

**DOI:** 10.1186/s40644-025-00823-x

**Published:** 2025-01-30

**Authors:** Johanna Ingbritsen, Jason Callahan, Hugh Morgan, Melissa Munro, Robert E. Ware, Rodney J. Hicks

**Affiliations:** 1 Melbourne Theranostic Innovation Centre, Level 8, 14-20 Blackwood St, North Melbourne, VIC 3051 Australia; 2 Department of Medicine, The University of Melbourne, St Vincent’s Hospital, Melbourne, VIC Australia

**Keywords:** Total-body PET, FDG, Image quality, Acquisition parameters, Radiation dose

## Abstract

True total-body and extended axial field-of-view (AFOV) PET/CT with 1m or more of body coverage are now commercially available and dramatically increase system sensitivity over conventional AFOV PET/CT. The Siemens Biograph Vision Quadra (Quadra), with an AFOV of 106cm, potentially allows use of significantly lower administered radiopharmaceuticals as well as reduced scan times. The aim of this study was to optimise acquisition protocols for routine clinical imaging with FDG on the Quadra the prioritisation of reduced activity given physical infrastructure constraints in our facility. Low-dose (1 MBq/kg) and ultra-low dose (0.5 MBq/g) cohorts, each of 20 patients were scanned in a single bed position for 10 and 15 min respectively with list-mode data acquisition. These data were then reconstructed simulating progressively shorter acquisition times down to 30 s and 1 min, respectively and then reviewed by 2 experienced PET readers who selected the shortest optimal and minimal acquisition durations based on personal preferences. Quantitative analysis was also performed of image noise to assess how this correlated with qualitative preferences. At the consensus minimum acquisition durations at both dosing levels, the coefficient of variance in the liver as a measure of image noise was 10% or less and there was minimal reduction in this measure between the optimal and longest acquisition durations. These data support the reduction in both administered activity and scan acquisition times for routine clinical FDG PET/CT on the Quadra providing efficient workflows and low radiation doses to staff and patients, while achieving high quality images.

## Introduction

PET/CT imaging using [^18^F]-fluoro-2-deoxyglucose ([^18^F]-FDG) is now an established modality for tumour localisation, staging and monitoring treatment responses, but its diagnostic accuracy heavily depends on image quality [1]. Scanner performance is dependent on the sensitivity and reconstruction algorithm of the system, the administered activity of radiopharmaceutical, and the duration of scan acquisition [2]. Recent advances in PET/CT systems, particularly including introduction of digital PET detectors, have improved technical specifications [3]. Digital PET systems replace standard photomultiplier tubes with silicone photomultipliers (SiPMs), improving spatial resolution, sensitivity and time-of-flight (TOF) capabilities [3, 4]. The enhanced characteristics of digital PET/CT systems support increased diagnostic performance over analogue PET/CT scanners, primarily by increasing signal-to-noise ratios [3].

However, system sensitivity is also affected by the length of the axial field-of-view (AFOV) [5]. A conventional AFOV PET/CT system typically covers only 15-25cm of the body at any time [6]. As the radiation is emitted isotropically, only 1–3% of the annihilation events emitted from a patient produce a detected line of response (LOR) in such systems [5, 6]. A “whole-body” scan created from multiple overlapping images or by moving the patients continuously through the scan consequently inefficiently collects annihilation events and limits the number of LOR available for image reconstruction [5]. Extending the AFOV will increase the number of detected events and increase the sensitivity and signal-to-noise ratio of the system [6]. “Total-body” PET/CT systems with an AFOV of 1-2m have increased system sensitivity by a factor of 10–40 over a conventional AFOV scanners and open the potential for reducing scanning times, administered activities or both [7, 8].

The Siemens Biograph Vision Quadra combines several such technical advances. It is a digital PET system with 3.2mm LSO crystals interfacing with SiPMs, providing high spatial resolution and TOF performance, and has 4 rings of 26cm detector blocks, extending the AFOV to 106cm. This provides an 8 to tenfold increase in sensitivity over a conventional AFOV PET/CT scanner using a single ring of the same digital detector technology [6]. Importantly, the long AFOV allows coverage of all major organs simultaneously, shortening scan acquisition times and facilitating dynamic biodistribution studies [5]. Multiple publications [9–11] anticipate that extended AFOV PET/CT system such as the Siemens Biograph Vision Quadra could alter clinical practice wherein image quality could be maintained or improved despite significantly reducing administered radiopharmaceutical activities.

Reducing administered activity of tracers could serve to lessen patient anxiety related radiation exposure from PET/CT, while also allowing for PET scans to be performed in clinical indications where individual patient radiation sensitivity may limit optimal use. There are additional potential research advantages for the development of novel radiopharmaceuticals [12, 13]. However, these potential benefits must be balanced against longer scanning times that increase the likelihood of movement in the images due to patient discomfort or pain [1] which degrade image quality independently of the inherent quality of the imaging system. Furthermore, the time spent by each patient on the scanner and the administered activity to patients has significant implications for departmental workflows and design.

In our department, structural engineering, radiation protection regulations and space allocation for tracer uptake rooms created an imperative for minimizing the administered activity to each patient. As a research and development facility, we were also attracted by the potential of high-quality imaging with low administered activities allowing, for example, comparison of more than one agent in normal volunteers. Conversely, financial modelling also mandated adequate patient throughput to offset the relatively high capital costs of this scanner compared to a conventional AFOV scanner and allocating scanner time efficiently for positioning and acquisition of scans was an important consideration for workflows. The aim of our study was, thus, to determine the optimum imaging times for routine clinical studies performed on the Siemens Biograph Vision Quadra using low (1MBq/kg) and ultra-low (0.5MBq/kg) [^18^F]-FDG administered activities. In a real-world setting, patient comfort and scan tolerability, which benefit from shorter scanning times, must accommodate the reading clinician’s tolerance for image noise with uncompromised lesion detectability.

## Methods

This retrospective analysis was performed of patients injected with either 1MBq/kg or 0.5MBq/kg of [^18^F]-FDG and scanned on the Siemens Vision Quadra as part of a clinically indicated scan. The initial acquisition times were chosen empirically based on published data using an administered activity of 3.5 MBq/kg [9]. This retrospective study was approved by a professionally convened human research ethics committee (reference number: 2023–12–1561).

The Low dose group consisted of 20 patients who were injected with 1MBq/kg of [^18^F]-FDG (range of 0.9–1.1MBq/kg) and were scanned for 10 min for a single ultra-extended FOV PET bed. The list-mode PET data were reconstructed with the following acquisition timing: 10, 5, 4, 3, 2, 1 and 0.5 min.

The Ultra-low dose group consisted of 20 patients who were injected with 0.5MBq/kg of [^18^F]-FDG (range of 0.5–0.6MBq/kg) and were scanned for 15 min for a single ultra-extended FOV PET bed. The list-mode PET data were reconstructed with the following acquisition timing: 15, 10, 6, 5, 4, 2 and 1 min.

All images were reconstructed using Siemens proprietary software, TrueX + TOF (ultraHD-PET), with a 5mm Gaussian filter, 4 iterations and 5 subsets. Each correlative CT for attenuation correction was performed using a tin filter, CAREkV and ADMIRE reconstructions to reduce the effective dose from the CT component. A coefficient of variance (COV) in liver activity was calculated using a 20mm volume of interest in the right lobe.

An upgrade on the Quadra was installed which included an Ultra-High sensitivity (UHS) reconstruction mode that increases the acceptance angle of detected events to include the entire length of the detectors. This mode is also known as MRD322. In NEMA phantom studies using this full acceptance angle increases the sensitivity by a factor of two compared to high sensitivity (HS) pre-update reconstructions [14]. We reconstructed each of the patient’s 7 image sets in both groups with the UHS reconstruction and repeated the quantitative analysis.

### Qualitative image analysis

**Fig. 1:**
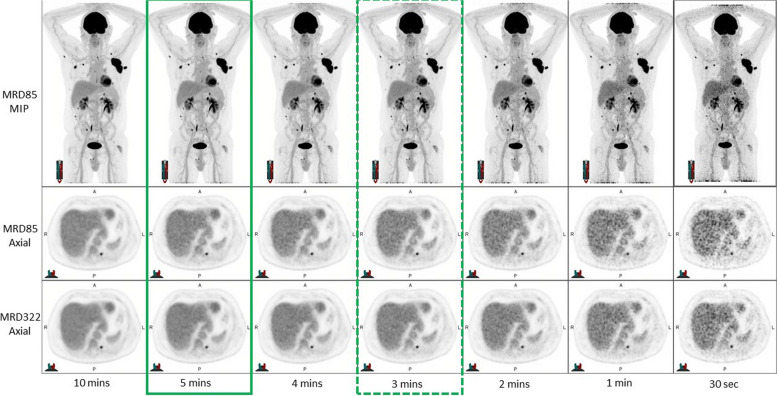
7 data sets for qualitative analysis – Low dose (1MBq/kg) The optimal time per frame is outlined with a solid green box and the minimal acceptable time per frame is outlined with a dotted green box based on the MRD85 reconstruction

**Fig. 2:**
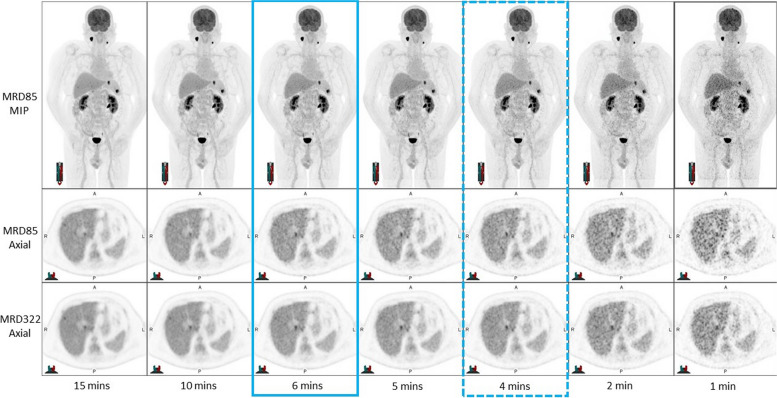
7 data sets for qualitative analysis – Ultra-low dose (0.5MBq/kg) The optimal time per frame is outlined with a solid blue box and the minimal acceptable time per frame is outlined with a dotted blue box based on the MRD85 reconstruction

All image sets were independently reviewed by two Nuclear Medicine Physicians, each with more than 20 years of experience in reading PET studies, to assess: overall image quality, lesion detectability, and diagnostic reporting preference. This was performed by displaying all reconstructions from longest to shortest acquisition time in a single display for each individual patient as shown in Figs. 1 and 2 for 1 MBq/kg and 0.5 MBq/kg respectively.

For each patient, the reader was asked to nominate the following:

- Minimum scan time—The minimum acceptable time was defined as the shortest scan where the images would be considered diagnostic and no clinically significant finding would be missed.
- Optimal Scan time—The optimal time was defined as the acquisition time that produced high quality images where there was no significant perceived benefit in image quality from a longer acquisition, with the implicit goals of maximizing in patient comfort and departmental workflows. For each patient, the longest time per frame was considered the gold standard against which comparisons were made.

### Quantitative image analysis -coefficient of variation (image noise)

For both the Low Dose and Ultra-Low Dose groups, quantitative analysis was performed using MIM Encore (MIM software Inc, Version 7.2). 20mm 3D contours were placed in the liver for each individual patient with contours matched across the patient’s series of reconstructions.

From these contours, the SUV_mean_ (standardised uptake value mean) and SUV_sd_ (standardised uptake value standard deviation) was recorded for each reconstruction timepoint and used to calculate the coefficient of variation for the liver to indicate the level of noise in the reconstruction. The coefficient of variation (COV) is the ratio of the standard deviation to the mean.

### Effective dose

For each individual patient, the effective dose from the [^18^F]-FDG injection and the DLP from the CT scan was calculated by the Siemens Software and recorded. The mSv effective dose from the CT was calculated by multiplying the DLP with the _k_factor for torso of 0.015. These effective doses were averaged over both the 0.5MBq and 1MBq cohort to give an average total whole-body radiation dose for a hemi-body PET/CT scan.

## Results

### Qualitative review

An example of the images at low and ultra-low injected activities are shown in Figs. 1 and 2. There is decreasing noise in the liver and other tissues with increasing acquisition time but lesions are clearly visualized even at short acquisition times in these examples. Table 1 shows the results of the qualitative review across both patient cohorts. The qualitative review of the Low dose group image sets resulted in an average minimum time per scan of 2.6 min with an optimal time per scan of 3.3 min. The qualitative review of the Ultra-low dose group images sets resulted in an average minimum time per scan of 4 min with an optimal time per scan of 5.6 min. To accommodate the preferences of the reader who was least tolerant of noise (Reader 2), we elected to use 1.0 MBq/kg and set minimal and optimal acquisition times of 3 and 5 min, respectively, for routine clinical studies unless clinical circumstances warrant minimizing radiation dose in which case would use 0.5 MB/kg and use minimal and optimal acquisition times of 4 and 6 min, respectively.

**Table 1:**
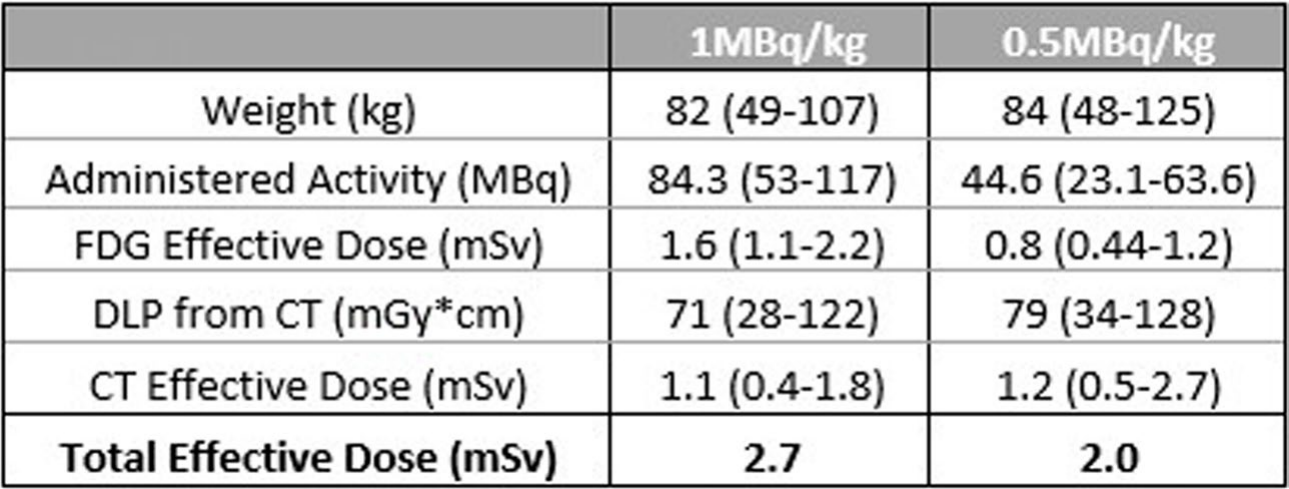
Reader Results for Low dose (1MBq/kg) and Ultra-low dose Images (0.5MBq/kg)

### Quantitative review

**Fig. 3:**
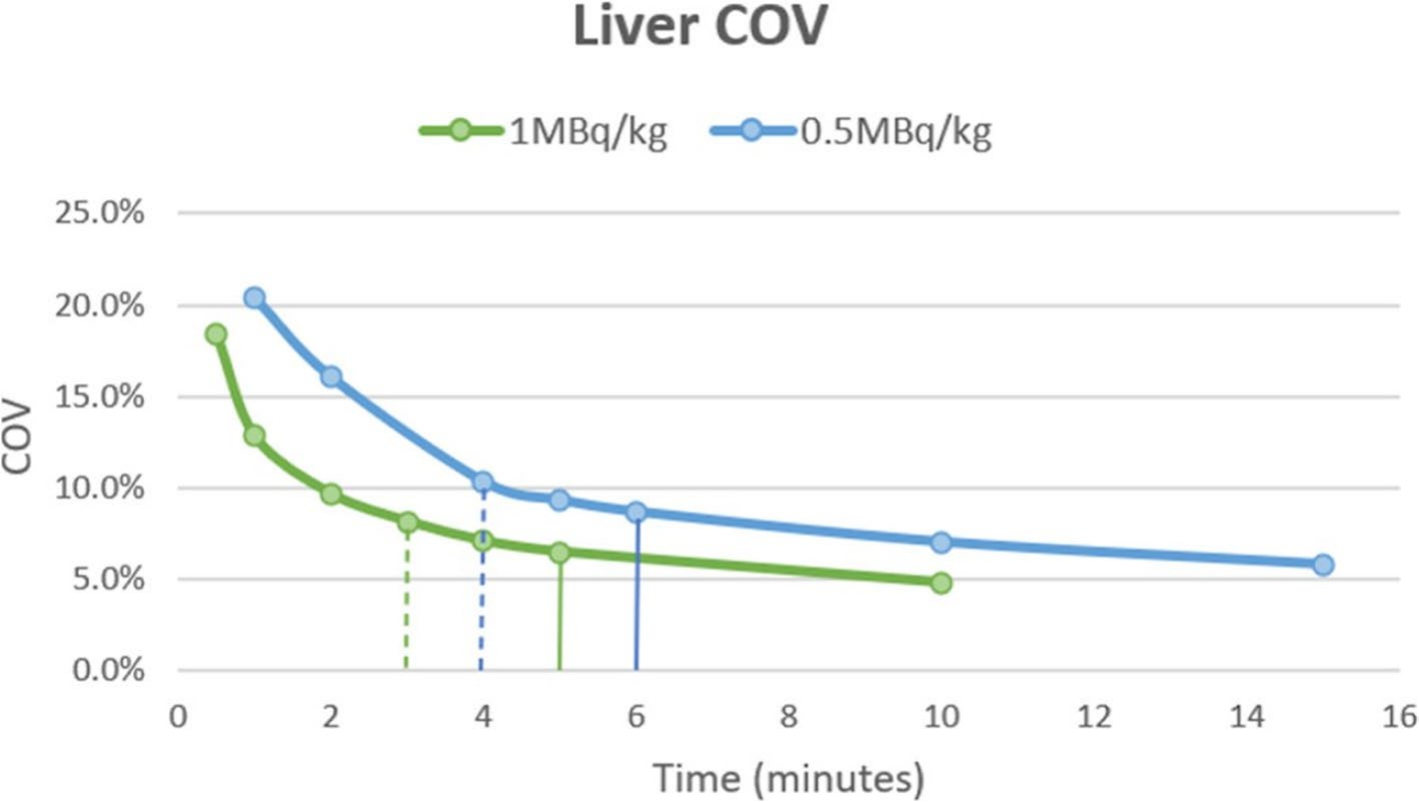
Liver COV (coefficient of variation) for the Low dose (1MBq/kg) and Ultra-low dose groups (0.5MBq/kg). The solid lines indicate the COV at the optimal acquisition time and the dashed lines indicate the COV at the minimum acquisition times

The results of the measurement of COV are shown below in Fig. 3. For the Low dose group (1MBq/kg) the COV for the liver for the physician-defined qualitative minimum scan time of 3 min was 8.1% and 6.5% for the optimum scan time of 5 min. For the Ultra-low dose group (0.5MBq/kg) the COV for the liver at the minimum time of 4 min was 10.3% and 8.6% for the optimum scan time of 6 min.

Increasing the scan times past the optimal time point in both groups did not yield a significant reduction in the COV. However, reducing the scan times below the minimum acceptable time per scan resulted in a significant increase in the COV, consistent with the reduced image quality perceived by expert PET readers. The COV for the liver from both cohorts at even the minimum acquisition time was well below the recommended EARL criteria of below 15% for image noise.

At the consensus optima and minimum acquisition durations, the coefficient of variance in liver was 10% or less for both administered.

### Ultra-high sensitivity scanning mode

**Fig. 4:**
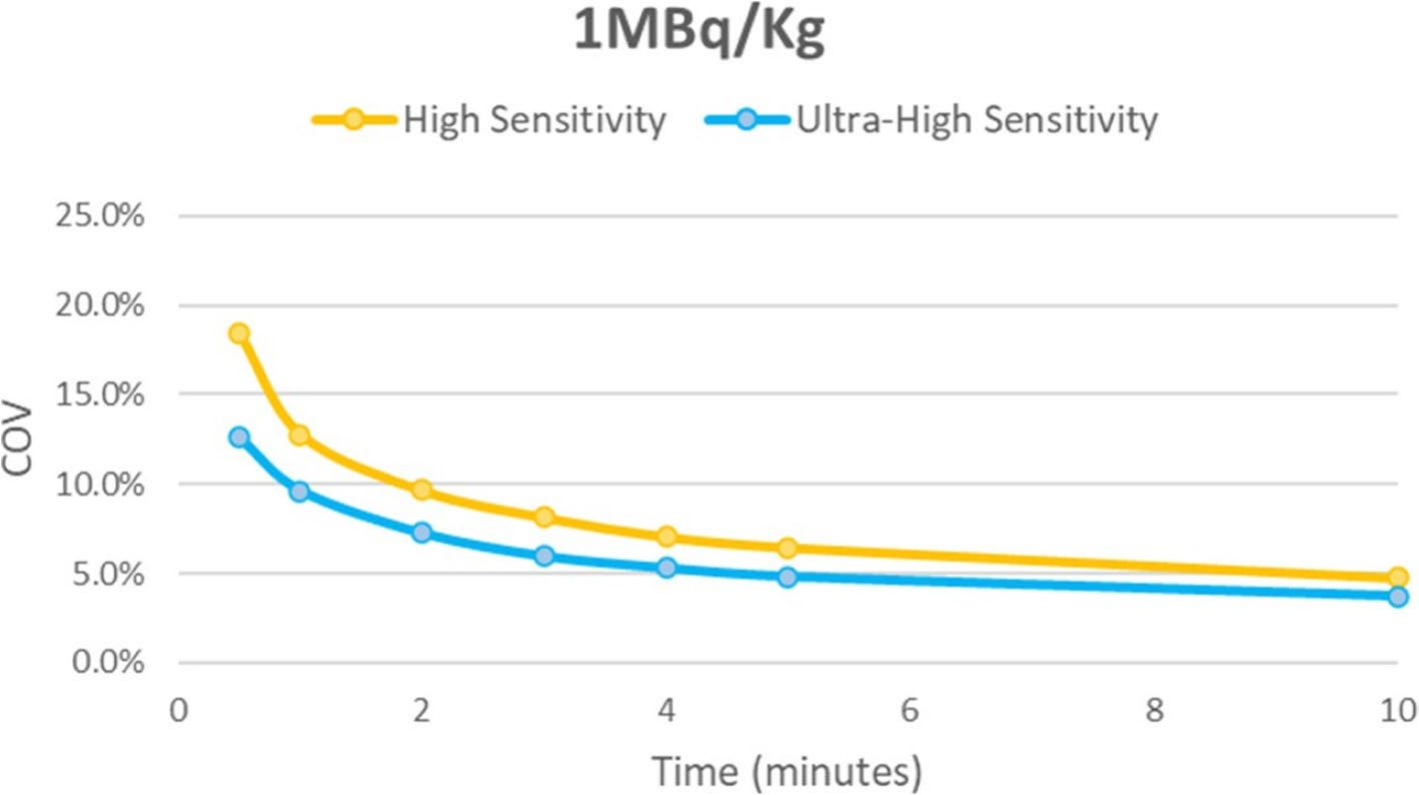
Coefficient of variation for the low dose group for High sensitivity (MRD85) and ultra-high sensitivity (MRD322) scanning modes using 1MBq/kg

**Fig. 5:**
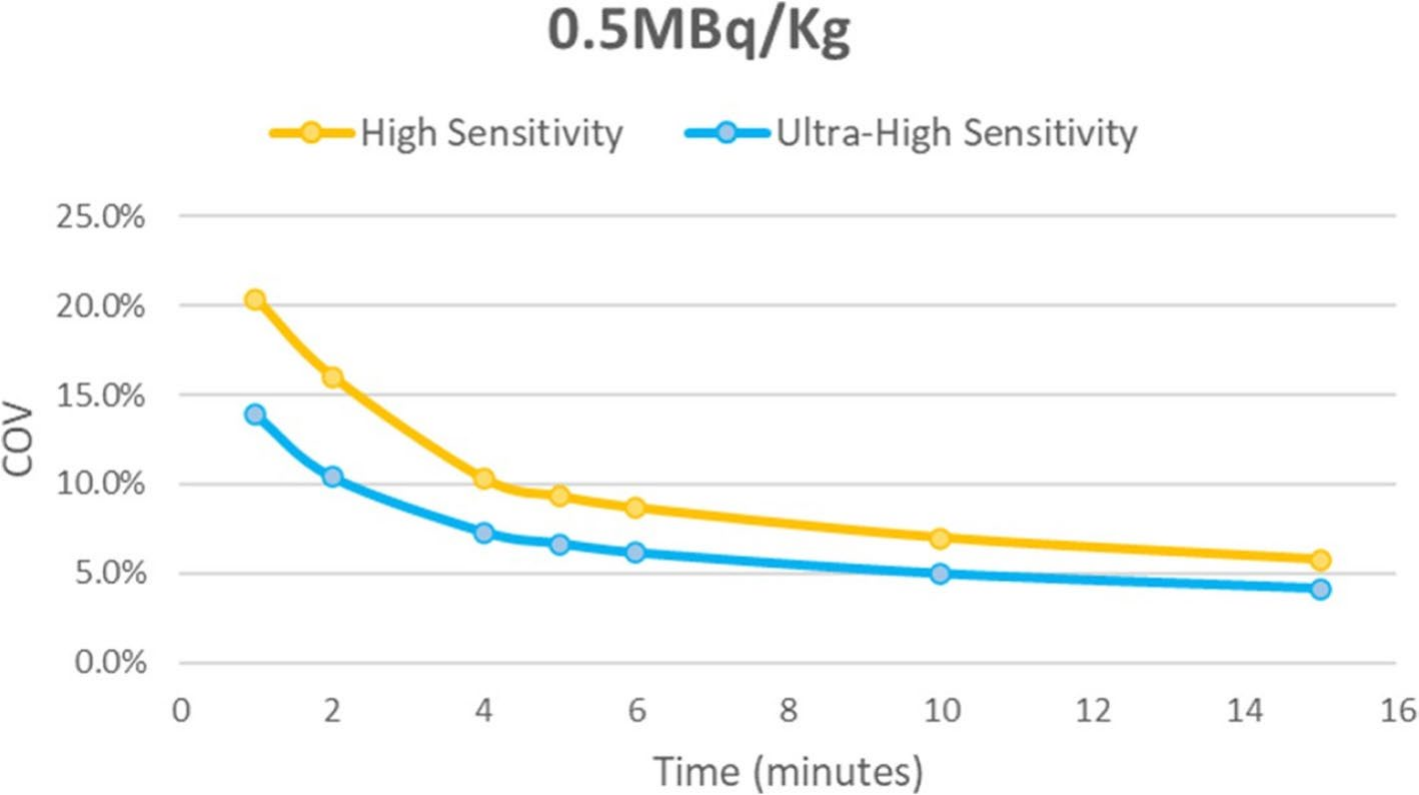
Coefficient of variation for the ultra-low dose group for High sensitivity (MRD85) and ultra-high sensitivity (MRD322) scanning modes using 0.5MBq/kg

As can be seen in Figs. 4 and 5, the UHS mode resulted in a reduction in the image noise as measured by the liver COV, particularly at shorter acquisition times, potentially allowing for minimum acquisition times of around 2 min or less, depending on administered activity while maintaining a hepatic COV < 15%.

### Effective dose

A summary of the administered activities and estimated effective doses is provided in Table 2.

**Table 2:**
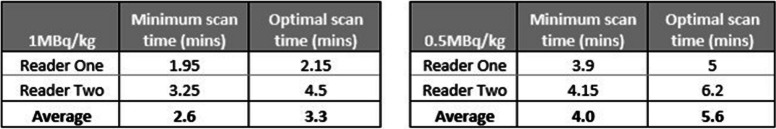
Average weight, administered activity and dose data for Low dose (1MBq/kg) and Ultra-low dose groups (0.5MBq/kg)

For the Low dose group, the patients received an average administered activity of 84.3MBq of [^18^F]-FDG with an average weight of 82kg (1.02MBq/kg). The effective dose calculated for [^18^F]-FDG by the Siemens software gave an average dose of 1.6mSv with the average effective dose from the CT of 1.1mSv giving a total effective dose of 2.7mSv for the procedure.

For the ultra-low dose group, the patients received an average administered activity of 44.6MBq of [^18^F]-FDG with an average weight of 84kg (0.53MBq/kg). The effective dose calculated for [^18^F]-FDG gave an average dose of 0.8mSv with the average effective dose from the CT of 1.2mSv giving a total effective dose of 2mSv for the procedure.

## Discussion

These data show that the increased sensitivity of extended field of view PET scanners allows for a reduction of both scan time and injected activity without compromising scan quality. With higher injected activities an even faster scanning time would be possible, which is especially valuable for poorly compliant patients unable to remain still, those with significant pain or who suffer claustrophobia. However, from a workflow perspective, there is little practical difference between a 2 min and 5-min scan when allowing for the time taken to get a patient on and off the scanner.

There are many benefits to the use of lower administered activities. The use of administered activities of 0.5MBq/kg or 1MBq/kg in clinical practice can have multiple areas of impact for a department and its patients. Lowering the activity administered to a patient during a PET scan will reduce the patient's exposure to ionizing radiation, maintaining ALARA principles to limit their lifetime exposure to radiation [11, 15]. This is particularly significant for paediatric patients who receive multiple PET/CT scans throughout their treatment and follow up process. Anxiety experienced by some patients over radiation exposure from imaging procedures may be eased knowing that they will be exposed to a significantly reduced amount of radiation.

The lower administered activities will also reduce the exposure to staff when preparing and administering injections, getting the patients on and off the scanning bed and when de-cannulating patients. The overall throughput of the scanner can also be increased as individual patient scan times can be reduced from ~ 25 min on a single FOV scanner to 5 min using an extended AFOV scanner. A reduction in administered activity also has implications for the design of the departments shielding requirements. The lower activities and faster scans will lead to a reduction in the amount of shielding required to be installed. In addition, the amount of activity that is needed to be provided by a radiopharmaceutical supplier is less, reducing the burden on the supplier to provide enough dose and therefore potentially decreasing the cost of the radiopharmaceutical.

The reduction of radiation exposure using the Ultra-low dose protocol is also desirable when performing PET scans on research participants or vulnerable population such as paediatric patients and pregnant patients [16, 17]. The ultra-low dose protocol is ideally suited for patients in these cohorts with an estimated absorbed dose of around 2 mSv which is equivalent to the annual background exposure of the general public [18].

Scanning paediatric patients using a balanced combination of reduced radiation exposure and reduced acquisition times on a large AFOV scanner can significantly benefit the patient while maintaining a high diagnostic signal-to-noise ratio of the images [10, 16]. Additionally, reducing the acquisition time may lower patient distress and eliminate the need for patient sedation, reducing the risks associated with anaesthesia, particularly if patients require multiple studies [16]. While an MRI component of a PET/MRI scanner does not contribute to a patient’s radiation exposure, the ability to significantly reduce the administered activity means that the overall radiation exposure from a PET/CT scan on an extended AFOV scanner may be less than on current generation PET/MRI devices [19].

For pregnant patients, the diagnostic benefit of performing a PET study for the initial staging of cancer is often weighed against the risk of foetal radiation exposure [17, 20]. This risk can be mitigated by lowering the administered activity using an ultra-low dose protocol, increasing hydration and encouraging frequent bladder emptying, thereby lowering the foetal exposure while maintaining diagnostic image quality to guide treatment decisions [20].

Patient and physiological organ movement during PET scanning also has a negative impact on image quality. Reducing the overall scan time can reduce movement artefacts in the images while also making the scan more tolerable for patients.

### Limitations

While programs such as the EARL criteria have been introduced to harmonise protocols across institutions with regards to quantitation there remains significant variation between institutions in acquisition time, injected doses, and reconstruction parameters based on qualitative image preference [21]. The main limitation of this study is that the qualitative evaluation of the images used to decide optimal and minimum scan times at the low and ultra-low doses were determined by highly experienced PET readers and may not be generalisable. Different reader preferences may make it necessary to repeat this optimisation process for low and ultra-low dose protocols at individual institutions before adopting routine scan acquisition durations. However, quantitative data suggest that the chosen times coincided with rapidly increasing noise. Additionally, this study gives confidence that such an optimisation process can be undertaken at significantly lower administered doses than are routine in short AFOV scanners without risk of unacceptable clinical quality scans occurring.

The increased acceptance angle allowed using UHS reconstruction gives a non-uniform sensitivity profile over the AFOV unlike the HS setting which is uniform throughout the FOV [14]. Accordingly, sgnal-to-noise ratio may differ at the edge of the FOV but was not investigated in this study.

## Conclusion

Guided by qualitative review of image quality by expert PET readers in a real-world clinical environment, this study demonstrates that it is possible to reduce both administered activities of [^18^F]-FDG and scan acquisition times while maintaining a low level of noise in scans performed on the Siemens Biograph Vision Quadra as part of routine clinical imaging. Image noise was further reduced by the introduction of the Ultra-High sensitivity mode, which presents opportunities for a further reduction in radiation exposure and even shorter scan times. These findings have important implications for clinical practice, workflow, and patient experiences. The potential benefits for research applications are also substantial.

## Data Availability

No datasets were generated or analysed during the current study.
